# Palpation force modulation strategies to identify hard regions in soft tissue organs

**DOI:** 10.1371/journal.pone.0171706

**Published:** 2017-02-15

**Authors:** Jelizaveta Konstantinova, Giuseppe Cotugno, Prokar Dasgupta, Kaspar Althoefer, Thrishantha Nanayakkara

**Affiliations:** 1 School of Engineering and Materials Science, Queen Mary University of London, London, United Kingdom; 2 Department of Informatics, King’s College London, London, United Kingdom; 3 AMRC Centre for Transplantation, DTIMB and NIHR BRC, King’s College London, Guy’s Hospital, London, United Kingdom; 4 Dyson School of Engineering, Imperial College London, South Kensington Campus, London, United Kingdom; Semmelweis Egyetem, HUNGARY

## Abstract

This paper presents experimental evidence for the existence of a set of unique force modulation strategies during manual soft tissue palpation to locate hard abnormalities such as tumors. We explore the active probing strategies of defined local areas and outline the role of force control. In addition, we investigate whether the applied force depends on the non-homogeneity of the soft tissue. Experimental results on manual palpation of soft silicone phantoms show that humans have a well defined force control pattern of probing that is used independently of the non-homogeneity of the soft tissue. We observed that the modulations of lateral forces are distributed around the mean frequency of 22.3 Hz. Furthermore, we found that the applied normal pressure during probing can be modeled using a second order reactive autoregressive model. These mathematical abstractions were implemented and validated for the autonomous palpation for different stiffness parameters using a robotic probe with a rigid spherical indentation tip. The results show that the autonomous robotic palpation strategy abstracted from human demonstrations is capable of not only detecting the embedded nodules, but also enhancing the stiffness perception compared to static indentation of the probe.

## Introduction

Palpation with a robotic device or artificial tactile exploration of soft viscoelastic and non-homogeneous objects is an important area of study for various fields such as medical and virtual reality applications [1–3]. In the scope of our attention is the implementation of tactile and haptic feedback for Robot-assisted Minimally Invasive Surgery (RMIS) that is typically performed using three-dimensional visual feedback only. In contrast, during open surgery, surgeon has direct access to internal organs and is able to perform soft tissue palpation to identify harder abnormalities, such as tumors. Several studies have underlined that the addition of tactile feedback during RMIS improves the performance of the surgeon and the clinical outcomes [4, 5].

The development of various stiffness and force sensing probes for RMIS [6] indicates the increased interest and the need for tactile systems during surgical applications. However, despite the active development of the field of medical tactile sensing, robotic palpation, or as named in this paper artificial palpation, is not yet implemented for RMIS. The detection of hard abnormalities and measurements from tactile devices—sensors that obtain tactile information from the target object—should be reliable and repetitive in order to be used for in-vivo medical applications. However, it is difficult to fulfill such requirements due to variability of conditions introduced by a surgical environment. That is caused by the viscoelastic nature of soft tissue as well as by external factors, such as movement of internal organs and flows of liquids.

The improved design of a tactile probe potentially can lead to better probing results. The current state-of-art research shows that various designs and transduction principles are employed and tested during the development of tactile devices for tissue examination [7–11]. Alternatively, it is possible to study manual palpation techniques that are broadly used during open surgery or physical examination to access the mechanical properties of the organs.

We propose that one of the possible directions is the development of behavioral guidelines in order to maximize the efficiency of probing devices during artificial tactile exploration. In our previous works [12, 13] manual palpation techniques were studied to understand the optimal control strategies that can be used by tactile devices to detect hard nodules inside a soft tissue more accurately. Two force-velocity modulation strategies of hard nodule detection were outlined [13]. The first strategy relies on the displacement and kinesthetic feedback perceived with the finger, while the second relies mostly on force feedback. These studies, inspired by manual palpation techniques, have shown the importance of the applied force and velocity modulations during unidirectional manual examination of a soft tissue. In the present work, it is important to explore the possible pattern of force modulation for the case of exploration of a given point. Therefore, the aim is to determine whether there exists a specific force control strategy that is used to detect harder areas in a soft environment.

Palpation techniques can be divided into three main strategies, according to the classification presented in [14], namely global examination, local examination and applied finger pressure. These techniques are often combined to achieve the best possible result. Global movement is applied as a general scanning and assessment technique. For further examination it is necessary to explore those areas more thoroughly. Therefore, the local finger movement technique is applied and performed only within a selected section. This type of palpation helps physicians to understand the shape and depth of an abnormality, and can be applied as tapping, sliding or vibration of the tissue. The third palpation technique, corresponds to the average intentional finger pressure applied during the palpation procedure, such as light and deep palpation [15]. Light pressure is mainly used with global scanning to access the general mechanical properties and temperature of the organs. Indentation of this type of palpation does not exceed 2 cm and pressure is as light as possible. Deep palpation is performed with heavier pressure, mainly used for local finger movement, with an indentation of about 4 to 6 cm, and is used to evaluate the stiffness, size, contours and shape of the formation or of the organ [16]. In this work we present studies on manual palpation that is focused on deep palpation for local examination patterns. Therefore, we look at the characteristics of force modulations applied for a single defined area.

The related studies of palpation techniques also indicate that to understand the mechanical properties or stiffness of soft harder objects one needs to apply specific examination behaviors. For instance, the importance of global examination technique in the initial object exploration is highlighted in [17, 18], and work in [19] discusses the effectiveness of force and depth control approaches during local palpation.

There are several examples when the properties of a soft medium are measured using active modulations of applied force, such as vibrations. One of the possible technologies to measure soft tissue stiffness during RMIS is a resonance-frequency based method. For instance, works in [20, 21] are based on the combination of linear variable differential transducers (LVDT) with mass-spring mechanism. The system measures a shift of accruing resonance frequency during the indentation of the probe inside a soft tissue. Thus, it is possible to obtain viscous and elastic properties of a soft tissue.

This paper focuses on understanding some of the important aspects of tactile exploration techniques applied during localized examination of a soft environment. In particular, we focus on local active tactile probing control strategies that involve force control strategies, and are used for one-point palpation or probing of a soft non-homogeneous environment. The knowledge and understanding of these behaviors can lead to the improvement of the palpation-based artificial tactile examination, as well as to contribute to the general understanding of force controlled robotic contact exploration. As part of the validation experiments for this work, we implement the autonomous palpation based on the obtained behaviors from human demonstrations. In this way, we validate the feasibility of the proposed methodology for remote tactile examination. The autonomous robotic tactile examination is a topic that becomes popular in recent years. For instance, work in [22] presents remote palpation using machine leaning approaches and studies in [23] describe the autonomous surface recognition using a vibration signal.

Based on the relevant literature and our previous findings, we can formulate the following questions to be discussed:

1. Whether there is a generic template or pattern of applied force that is used to probe a localized area of viscoelastic non-homogeneous environment;
2. Whether there exists a mathematical model that can represent the pattern of force modulation;
3. And consequently, whether a robotic probe of the mechanical design different from the shape of the human finger can achieve a substantial level of palpation effectiveness only by following the pattern found from human demonstrations.

Further on, Section II describes the methodology of palpation studies; in Section III the experimental results on soft tissue phantom palpation are presented. The force control strategies are implemented using autonomous robotic palpation in Section IV. Section V discusses the findings of the paper.

## Methodology

### Subjects and experimental protocol

In this paper we are particularly interested in understanding general force control principles for tactile exploration of soft environments, such as artificial palpation during RMIS. These studies aim to produce the results that can be generalized for various applications. The work carried out for this studies was approved by the King’s College London Biomedical Sciences, Dentistry, Medicine and Natural & Mathematical Sciences Research Ethics Subcommittee (BDM/11/12-84). Participants provided their written consent to participate in this study. The consent procedure was approved as part of the studies by the ethics committee.

In total we have obtained and analyzed 420 trials of palpation recording from seven palpation areas and twelve subjects performing five trials. Five out of twelve subjects had experience with clinical manual palpation. Previous research indicates that there might be a difference in palpation behavior between experts in palpation and novices [13, 24]. However, the above mentioned studies analyzed palpation applied across larger area involving finger displacement over the tissue. In this work, we also evaluate whether the applied dynamics of force modulation during palpation of the localized area is different for expert subjects.

**Fig 1:**
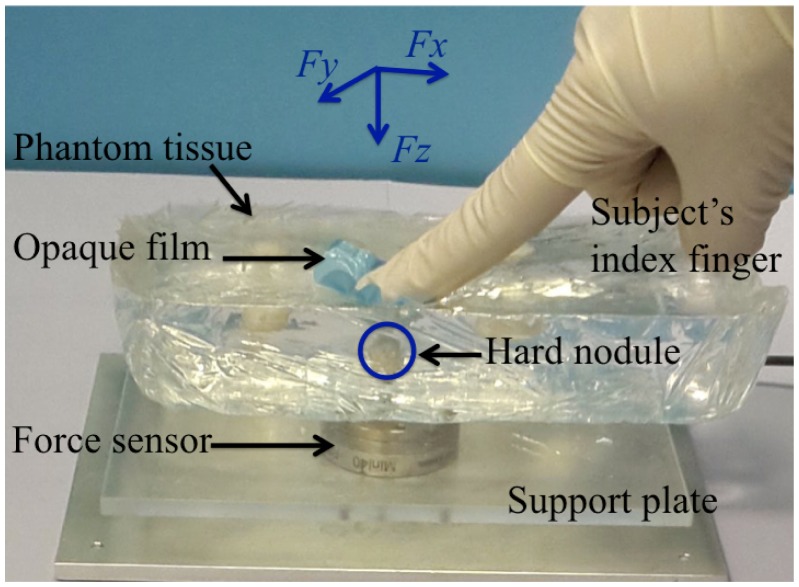
Experimental setting used for studies of local palpation behavior. Coordinate frame reflects lateral forces (*F*_*x*_ and *F*_*y*_) and normal force (*F*_*z*_) applied by subject’s index finger.

The participants were asked to explore the appointed areas of an artificial soft tissue phantom (Fig 1). Six out of seven areas contained an embedded nodule. During palpation experiments the area of a nodule was covered with an opaque film, and the target palpation area (10 mm^2^) was highlighted with a marker so that palpation movement is centered above the nodule. The film was made from textured double polymer coated latex that additionally helps to reduce unwanted sliding motion of the finger. Hard nodules of different diameters were fabricated to simulate tumors—3, 6, 9, 12, 15 and 18 mm. The palpation area with no nodule was used to study whether the absence of the nodule changes the behavior of force modulation during palpation. All the nodules are embedded on the same depth (5 mm) from the surface, as the perception of the tactile signal from different sizes and depths is coupled. A small nodule embedded close to the surface can produce the same signal as a large nodule embedded deep in the medium. In such way the results can be generalized for different depths as well. The sizes of nodules correspond to the cancer stage T1, according to TNM classification [25]. To prevent the effect of learning, subjects were asked to explore embedded nodules in a pseudo-random order. In addition, subjects were given time to get familiar with the task, performing three palpation trials on a different phantom tissue. Subjects were asked to estimate the depth and the diameter of an embedded nodule. This question was asked in order to stimulate people to apply the most effective natural exploration pattern using one finger palpation, and the answers given by subjects were not analyzed in the scope of this work. Subjects were instructed to palpate a single site on the silicone phantom, and to avoid sliding movements. They were free to explore the designated area by applying lateral and normal movements. It was observed that all subjects intuitively have used their index finger for palpation.

To comply with the common natural stiffness ratio in healthy tissue tumor, artificial soft phantoms with hard nodules were fabricated [26]. According to the previous studies [27] transparent silicone gel RTV6166 (Techsil Limited, UK) mixture ratio 4:6 and 900 mPa⋅s viscosity was used to create soft artificial tissue. Hard transparent nodules—artificial tumors—were embedded in the soft phantom tissue. To fabricate the spherical nodules, hard silicone rubber compound RTV615 (Techsil Limited, UK), ratio 10:1 and 4000 mPa⋅s viscosity was used. Four silicone phantoms, with two nodules of different diameter each, were created to study the forces applied during exploration movement, including the one that was used for the preliminary studies. The distance between two nodules of the same phantom was 50 mm. In case of a damage of the phantom or an internal displacement of the nodule, new silicone phantom was used.

### Data measurement

The experimental setup (Fig 1) was designed to measure the applied force during palpation of an artificial soft tissue. Three-dimensional force readings were recorded using 6 degrees-of-freedom force and torque sensor (Mini 40, ATI industrial automation). The resolution of normal force is 0.01 N and the sensing range in that direction is ± 30 N. The range of lateral forces is ± 10 N with resolution 0.05 N. The sampling frequency of the sensor is set to 1000 Hz. In order to smooth high frequency peaks, a moving average filter was used with the span of 21 data points that is equivalent to low-pass filter. The sensor was mounted under a support base plate that holds the phantom tissue. After the placement of the silicone phantom on the plate, accrued forces and torques were biased to zero. The sample of artificial tissue was placed in such a way that the area of examination is located just on the top of the six axis force and torque sensor. As the silicone phantom was transparent the alignment of the nodule was implemented visually, using the top view. As it is mentioned above, the area of the nodule was covered with an opaque film. During palpation, the silicone block was stably attached to the base under its own weight, and any sliding or bending was avoided.

To observe the variability of the force measurements for three force components across subjects and all trials, standard deviations of normalized values were analyzed. Measurements were normalized by the maximum value to the range from zero to one. For statistical analysis, a Kolmogorov-Smirnov test was used to check if the data is distributed normally. Analysis of variance (ANOVA) and two-way t-tests were used to test statistical significance of palpation factors that were considered significant, if the null hypothesis was rejected with a 95% confidence level (*p* <0.05). Repeated measures design of statistical analysis was applied for the obtained data, as it is used to evaluate different factors at the same time. Seven locations of palpation (different diameters of the nodules), as well as five trials performed by each subjects are within-subjects variables. The palpation experience of participants (experts and novices) is between subjects factor. The dependent variables that were tested are as follows: standard deviation of lateral and normal force, magnitude and frequency of lateral forces. Within-subject and between subject factors were always considered for the ANOVA test where it is applicable. A post hoc Tukey honest significance test was used for the correction of multiple comparisons.

## Analysis of the applied forces during palpation

### Variability of force magnitude

To achieve a better understanding of the ways humans detect hard nodules in viscoelastic environments, modulations of applied normal and lateral forces are evaluated separately.

**Fig 2:**
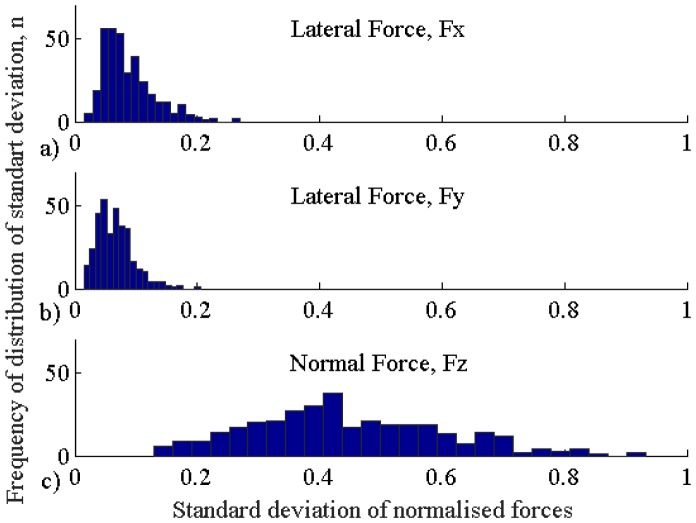
Histogram of standard deviations across all trials for normalized measurements. a) Lateral force *F*_*x*_, b) lateral force *F*_*y*_, and c) normal force *F*_*z*_.

Fig 2 reflects the histograms showing the variability of standard deviation values for three force components for all trials. Bin width for the histogram plots was selected based on Freedman-Diaconis rule. The magnitude of lateral forces (*F*_*x*_ and *F*_*y*_) is kept relatively at the same level with the average standard deviation *σ* = 0.04 (for normalized force values) and the mean magnitude of 1 N (for original force values). In addition, the deviation of lateral forces did not depend on the diameter of the nodule (*F*_3,416_ = 0.11, *p* = 0.73 for *F*_*x*_, and *F*_3,416_ = 0.43, *p* = 0.57 for *F*_*y*_). Conversely, the normal force has higher standard deviation (*σ* = 0.16) and the mean magnitude (3.2 N), and depends on the diameter of the nodule (*F*_3,416_ = 4.07, *p* <0.005) and subject (*F*_3,416_ = 5.23, *p* <0.0001), both for experts and novices. A post hoc analysis showed that the performance of one novice subject differs significantly (adjusted *p*-value: *p* <0.01); the influence of any diameter of the nodule is not significantly different from the other nodule. Therefore, this might suggest that subjects predominantly use normal force to search for hard nodules in soft tissue compared to lateral forces.

### Modulations of applied force

#### Lateral forces

**Fig 3:**
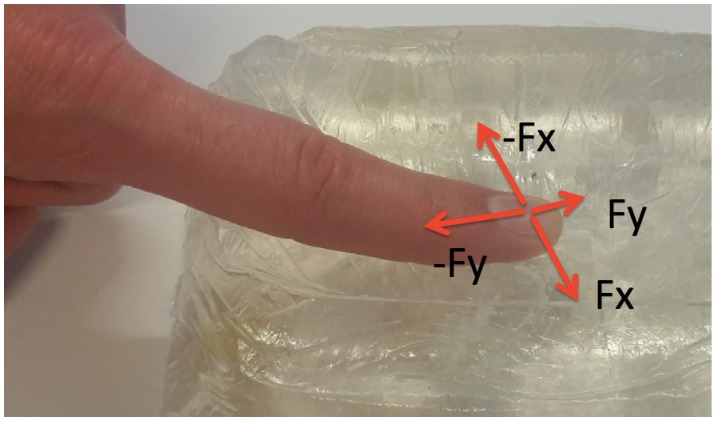
Histogram of correlation coefficients for measurements of two connected lateral forces Fx and Fy.

**Fig 4:**
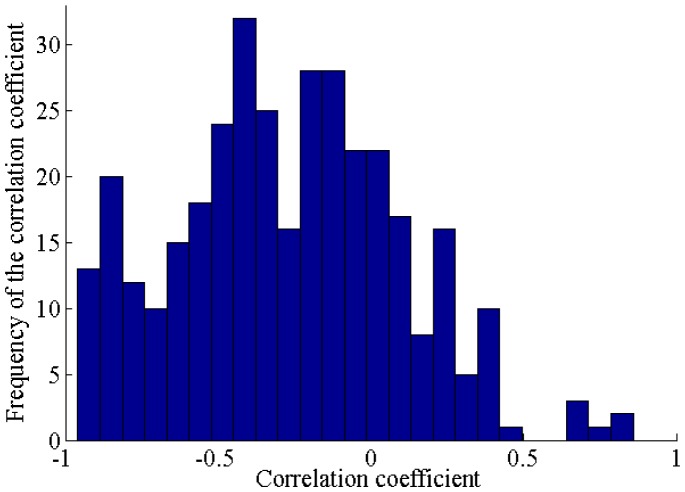
Constraints of the finger motion during single point palpation. For *F*_*x*_ (direction is chosen for this example), the finger can move freely in both directions, but in the case of *F*_*y*_ (direction is chosen for this example), the motion is limited by a finger joint.

To understand the possible patterns of modulation of lateral forces, *F*_*x*_ and *F*_*y*_ measurements were analyzed. These two forces are mechanically related as the force modulation is performed on the same plane. The correlation analysis was carried to support this interpretation. According to this analysis, 94% or 395 trials have significant correlation with mainly negative trends (p <0.05), as it is shown in Fig 3. This confirms the fact that the two forces are inversely related. The combined lateral force forms an ellipsoid because of the morphological constraints of a human finger. As it is shown in Fig 4, the motion is limited for one direction because of the distal joint of the finger.

**Fig 5:**
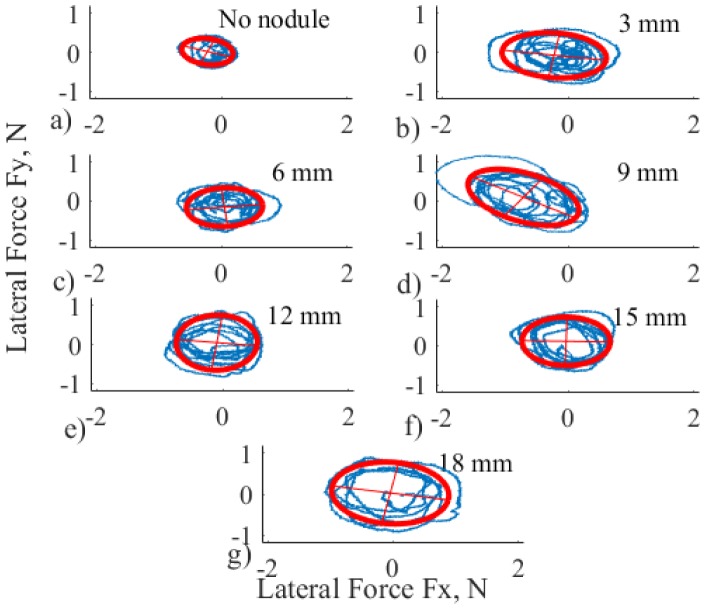
Distribution of lateral force with fitted ellipses (red dashed line) for one selected subject, one trial, for different nodules a) to g): Empty, 3, 6, 9, 12, 15 and 18 mm, respectively.

To characterize the magnitude of lateral forces in one variable, the area of a fitted ellipse (Fig 5) was used. Different combinations of semi-major and semi-minor axes can lead to the same area of an ellipse, but as both of the forces are correlated, this does not influence overall analysis. Fig 5 displays the distribution of magnitude of lateral forces for one selected subject and one trial. The fit of the ellipse was implemented using custom MATLAB function *ellipse_fit* [28] that is estimating ellipse parameters using least square method.

To check if it is possible to model the relationship between the nodule diameter and the magnitude of the lateral force, characterized by the area of an ellipse as a curve, linear regression analysis was used. According to the evaluation of the coefficient of determination for polynomials of different orders, the mean order of best fit is as high as 3.7 ± 1. In addition, the results of statistical analysis show that the magnitude of lateral forces is independent with respect of the nodule diameter and the preferences of the subject (*F*_3,416_ = 0.24, *p* >0.05, for both subject groups separately, and all subjects together).

**Fig 6:**
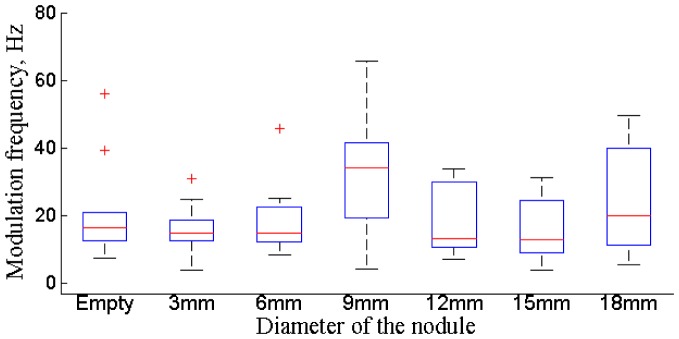
Distribution of frequency for the modulation of lateral forces for different probing locations for all subjects.

Lateral forces follow the sinusoidal pattern, and Fig 6 displays the distributions of the applied frequencies for all subjects. Frequency is calculated as a mean value for each trial. The applied frequency of the lateral forces is not influenced by subject preferences and experience (*F*_3,416_ = 0.81, *p* = 0.38), by the diameter of the nodule (*F*_3,416_ = 0.24, *p* = 0.66), or by the number of selected trial (*F*_3,416_ = 0.055, *p* = 0.81). Thus, it is possible to identify a specific bandwidth of frequencies for lateral forces that is used during exploration of the given environment that has the mean value of 22.3 Hz. It is possible to assume that there is a stereotypical force modulation frequency used by all subjects. The developed frequency of force modulation might occur due to the natural constraints of human hand ligaments in combination with the given viscloelastic environment [29].

#### Normal forces

Next step of the analysis is to understand whether the modulations of normal force are random or follow any specific pattern during the exploration. The relationship of normal force in respect to lateral is studied using correlation analysis. It was found that there is a significant negative correlation (mean value equals to −0.45) between the magnitude of lateral and normal forces (*p* <0.05 for 93% of all trials). This indicates that the probing strategy includes both modulation of lateral and normal forces. This is taken into account during the development of an autonomous robotic palpation, described later.

As it was observed in the previous sections lateral force follows fixed modulation, as there is no significant variation of lateral forces among subjects and across trials. Therefore, it is possible to conclude that the normal force can be modeled separately. Normal force can be the main force vector that is used to produce a stiffness feedback to detect an embedded hard formation. In the next subsection the mathematical representation that can be used to model the normal force is studied.

### Modeling of the modulations of normal force

**Fig 7:**
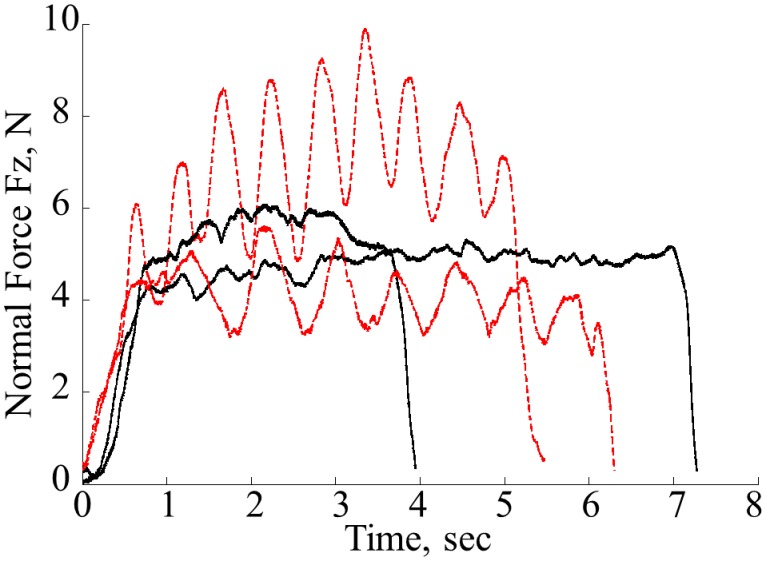
Sample profiles of normal forces for sinusoidal (red dotted lines) and step—like response (black solid lines) from diffident subjects.

Observations of the behavior of the applied normal force show two main patterns used by subjects—sinusoidal and ramp like modulation of the force with relatively short convergence time. It was noted that all subjects were consistent in the choice of the pattern for all trials for each nodule location. The sample profiles of normal forces observed from different randomly selected subjects using the two types of profiles are shown in Fig 7—sinusoidal is shown in red dotted lines and step-like is shown in black solid lines. Although, two separate patterns can be observed, the goal of this work is to determine whether it is possible to find one generic pattern that describes the modulations of normal force. Typically, human behaviors can be characterized using forward predictive models [30]. In addition, the empirical evaluation of data excludes the possibility of linear time-dependent modeling.

One option is to model the transient behavior as a Markov decision process with the steady state treated as an absorbing state. However, we note that the settling time *t*_*s*_ to an absorbing state in this case is just 3.3 seconds. The value was calculated using the second largest eigenvalue *λ*_2_ of the probability distribution matrix: *t*_*s*_ = 1/(1 − *λ*_2_) [31]. The settling time is too short to be modeled as a stochastic decision process. Therefore, we focus more on the steady state behavior in this study. For the validation of the mathematical model, the experimental data is split into 80% of training data and 20% of the validation data.

In this section both reactive and predictive models are tested. In the first case it is assumed that subjects are planning the applied stress using previously perceived information about stress values, while for the second case the estimated or predicted stress is used. The autoregressive model can be defined with the following equation:

$$\begin{array}{c} X_{t}=c+\sum_{i=1}^{n}a_{i}X_{t\pm i}+\varepsilon_{t} \end{array}$$ (1)

where *a*_*i*_ are the autoregression coefficients, *ε*_*t*_ is white noise, *c* is constant *X*_*t*_ is the stress output, *t* is the sampling step, and *n* is the model order. In our case the constant *c* is equal to zero. In case of (*t* + *n*) the model is predictive, in the case of (*t* − *n*)—reactive. To understand the order of the autoregressive model for both cases, the Akaike Information Criterion (AIC) was used. To statistically validate and choose the model order, non-parametric Wilcoxon rank sum test was implemented across AIC values for different orders. This test is equivalent to Mann-Whitney U-test and is used to test whether the rank of related samples is different. The AIC values were calculated per model across the entire validation data set. Pairing between model orders was used to evaluate the best model order number. The Table 1 below reflects *p*-values for reactive and predictive models for different orders. The high p-value between higher orders indicates low information gain for such change. Based on these results, it can be concluded that the modulation of normal force can be best described using either a second order reactive model (*p* <0.001), or a third order predictive (*p* <0.05).

**Table 1:** Selection of Order Number for Autoregressive Model.

*Order Number*	*Reactive model*, *p*-*value*	*Predictive model*, *p*-*value*
One vs. Two	*p* < *0.0001*	*p* < *0.0001*
Two vs. Three	*p* = 0.38	*p* < *0.05*
Three vs. Four	*p* = 0.76	*p* = 0.34
Four vs. Five	*p* = 0.84	*p* = 0.63
Five vs. Six	*p* = 0.98	*p* = 0.74

Further on, the coefficients of the autoregressive model were obtained. Stochastically distributed coefficients obtained from the individual fitting are the result of the learning phase. To estimate these values, centroids of the distribution of coefficients were used. The coefficients of the second order reactive model were estimated as: *a*_0_ = 1, *a*_1_ = −1.706, *a*_2_ = 0.721. The predictive model of the third order can be characterized by the following coefficients: *a*_0_ = 1, *a*_1_ = −1.980, *a*_2_ = 0.819 and *a*_3_ = −0.164. The autoregressive model was applied for both patterns, which are mentioned in the previous section—sinusoidal and step-like.

Two obtained models were tested on the validation data, using costs of normalized root-mean-squared error (NRMSE) as a measure of the goodness of fit. This criterion is a non-dimensional version of root-mean-squared error (RMSE). NRMSE allows to compare data of different dimensions dividing RMSE by the range of the observed data. The model produces a perfect fit if the cost criteria of the validation data is 100% and lower values indicate a decreasing fit. In this study, we use a threshold of 70% to determine an acceptable fit The relative comparison is used to select the appropriate model.

**Fig 8:**
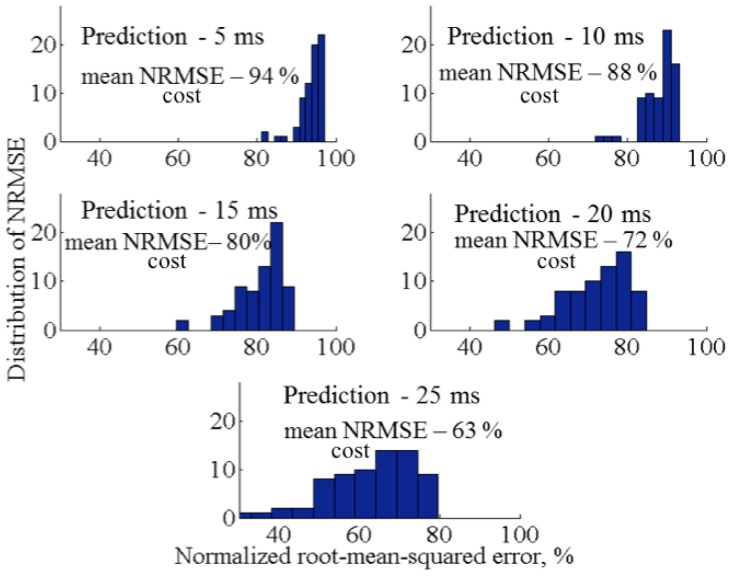
Histograms of prediction horizon and costs of NRMSE for second order reactive model, 100% assumes perfect fit.

It was found that reactive model produces a good fit up to 20 ms prediction horizon. Fig 8 displays the corresponding average cost values for reactive autoregressive model for prediction horizon 5 to 25 ms. The prediction horizon shown in this figure is calculated for the estimation of each next step. The frequency of the force data is 1000 Hz. Thus, each time frame is 1 ms long. On the other hand, a predictive model is not able to produce good fitness results. The average cost value for prediction horizon 5 ms is 95%. However, the increase of horizon results in bad fitting below 60%. Therefore, we can conclude that the data of normal force can be best explained with the second order reactive model.

## Robotic implementation of the algorithm for local palpation

To validate whether the obtained reactive model of force modulations can be used during artificial palpation, experiments were performed using a robotic setup. The goal is to verify if the human-like palpation can be replicated during robotic palpation, and if this type of artificial palpation can enhance the stiffness perception from the hard embedded nodules. In particular, it is important to verify this approach for exploration of the nodules that are difficult to perceive, such as very small or deep nodules. For the prediction of the behavior of the normal force, a second order predictive autoregressive model was used according to the findings of the human experiments.

### Experimental setup

**Fig 9:**
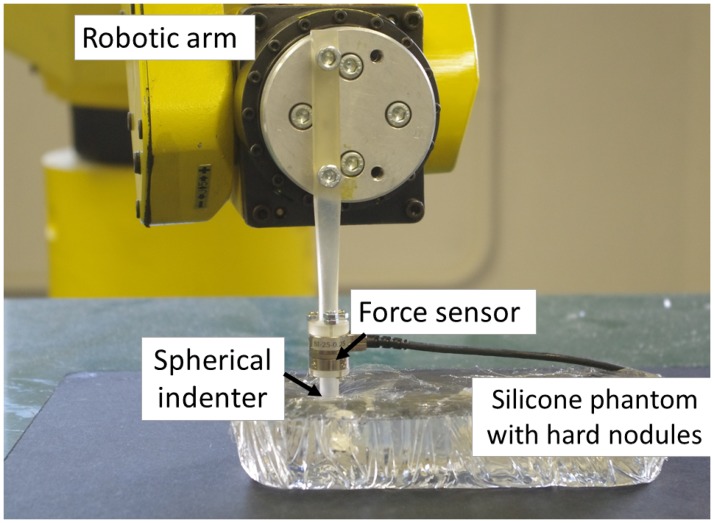
Experimental setup to validate autonomous palpation based on force modulation strategy.

To perform autonomous robotic palpation, a tactile probe was attached to the robotic arm Fanuc M-6iB with R-J3iB controller. The robot arm has 6 DoF and ± 0.08 mm repeatability of the motion. Tactile probe has a spherical indenter, 8 mm in diameter. The position of the probe is measured from the position of the end effector of the robot using forward kinematics. A commercially available force and torque sensor NANO17 (ATI industrial automation, force resolution 1/320 N) was used to measure applied forces during probing. The probe is positioned on the surface of the silicone in the desired location before palpation. The maximum possible indentation depth of the probe is 6 mm. The palpation algorithm autonomously controls the modulation of the applied normal force, but not the indentation depth. The safety threshold is needed to avoid breakage of the tissue or of the probe. In addition, it is required to preserve the linear elastic limit for the silicone phantom [32] to obtain correct stiffness values. Fig 9 displays the arrangement of the experiments.

### Design of validation experiments

In the presented work the aim is not to mimic human behavior, but to understand patterns of human palpation for one localized area, and subsequently to implement and to adapt the behavioral pattern for robotic implementation. The target of the validation experiments, described in this section, is to determine whether the outlined mathematical model of human behavior can be implemented in robotic applications. It is required to compare the performance of the proposed force-based modulation strategy with simple indentation-based or passive palpation, such as in [33], when the stiffness is measured after the indentation of the probe into material.

The Young’s or Elastic modulus is used to evaluate the perceived stiffness values during the autonomous palpation. It is reported [34] that for a spherical indentation tip and a small indentation depth, the Young’s modulus of soft tissue can be calculated as follows:

$$\begin{array}{c} E=\frac{3f(1+v)}{8d_{in}\sqrt{rd_{in}}} \end{array}$$ (2)

where, *E* is the Young’s modulus, *v* is the Poisson’s ratio, *f* is the normal interaction force, *r* is the radius of the indenter, and *d*_*in*_ is the indentation depth. Poisson’s ratio for the assumed incompressible soft tissue is 0.5.

For this experiment silicone phantoms with hard nodules of different diameters are used. Similarly to studies with human subjects the diameters were chosen to be 3, 6, 9, 12 and 15 mm, all embedded at a depth of 5 mm. Larger nodule is not considered, as the diameter of the indenter (8 mm) is approximately half size compared to the average width of the index finger’s fingertip that is 17 mm, according to reports in [35, 36]. Therefore, the robotic identification is performed both for nodules that can be difficult for humans to sense and that are easier to perceive. In addition, to evaluate perception between harder areas and soft uniform environment, the stiffness of the location with no nodule is evaluated as well. The validation experiments are performed using the following steps:

1. First, it is required to obtain the stiffness of each test location during static probing. The static probing or indentation-based palpation means that the probe is indented 5 mm down into the chosen location on the silicone phantom. The force is measured at this point and the corresponding stiffness is calculated. We call this measurement indentation-based stiffness. These stiffness values are used to validate the performance of the proposed autonomous palpation.
2. In the second stage of the experimental studies, autonomous palpation is performed on the same areas, that is, on five nodules and on empty locations. Five trials were performed for each separate location. This type of behavior is based on human tactile demonstrations and is formulated with the help of an mainly used for autoregressive (AR) model.
3. The stiffness measurements from the same locations are compared, and it is evaluated whether the proposed model can be used for robotic applications.
4. Finally, the validation of our modeling approach for the autonomous palpation is performed in Section. Robotic palpation is performed using modulations of lateral and normal forces separately.

### Control algorithm

The design of the control algorithm for autonomous palpation was based on the proposed experimental design. The position control of the robot is implemented based on the force feedback measured by the probing device.

The first stage of the algorithm is initialization of the system. The system is running with the time interval of 120 ms, that was chosen empirically. This time allows the system to reach the force defined by the autoregressive predictive model. The second step is the definition of the stiffness threshold that is used to determine the presence of the nodule. The stiffness threshold is calculated during the first indentation of the probe in the phantom organ. Finally, the second order AR model requires two force inputs to be initialised. Therefore, two first force readings are recorded for two indentations of 0.25 mm depth.

After the initialisation is complete, the palpation loop is started. According to human demonstrations, lateral and normal motions are applied also for robotic palpation. The sinusoidal modulation is created due to lateral motion with the range of 2 mm, and it is set to fluctuate at frequency of 22.3 Hz. Normal motion of the robotic probe is applied based on the desired force that is set by the AR model.

Each iteration of the palpation loop consists of the following steps: 1) the system obtains force readings from the tactile probe; 2) the desired palpation force is predicted according to the output of the AR model; and 3) the desired force is translated into normal displacement of the probe. The final stage is implemented via decremental or incremental indentations of 0.25 mm, and at every indentation it is checked whether the measured force corresponds to the desired force (with the tolerance of 0.01 N). In case the desired force is not reached within the set limit of 120 ms, last measured force value is used for the prediction with the AR model. The stiffness value is calculated according to Eq 2 at the end of each iteration.

**Fig 10:**
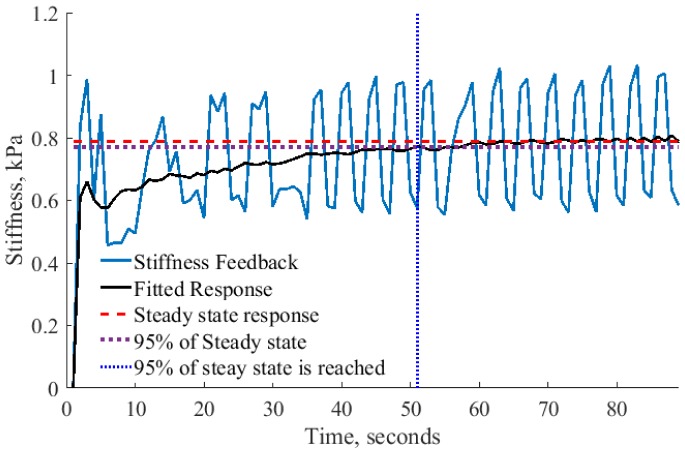
Example stiffness measurement for autonomous palpation. Analysis of steady state response to calculate the average time required to perform autonomous palpation.

Finally, it is required to understand the required length of robotic palpation to record the modulation of stiffness after the stiffness threshold is achieved. It is difficult to relate the speed of human palpation with the speed of robotic system. Temporal resolution of human touch with two successive stimuli is about 5 ms [37]. In addition, human decision making process regarding the presence of the nodule can depend on various external factors. Therefore, in the robotic implementation it is required to define a criterion determining the length of autonomous palpation and decision making process. It was observed (Fig 10), that after some time of palpation, the measured force feedback settles down to a steady state with vibrations. In the example in Fig 10, the black solid line shows a trend of the response without an oscillatory component that reaches 95% of steady state response at 51 seconds. The average time for all recorded trials to reach 95% of steady state is 55 seconds. This time was selected to evaluate the performance of robotic palpation. In addition, we have tested the robustness of the system for lower percentages to reach steady state response, but only at 95% the result was stable across all nodules.

### Analysis of stiffness measurement for autonomous robotic palpation

**Fig 11:**
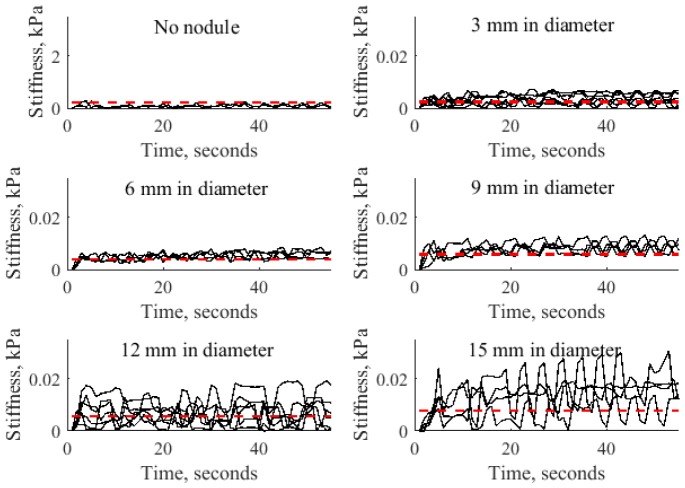
Stiffness measurements for indentation based measurements (red dotted line) and autonomous palpation based on AR model (solid line) for silicone with no nodule, nodules of 3, 6, 9, 12 and 15 mm for all trials.

**Fig 12:**
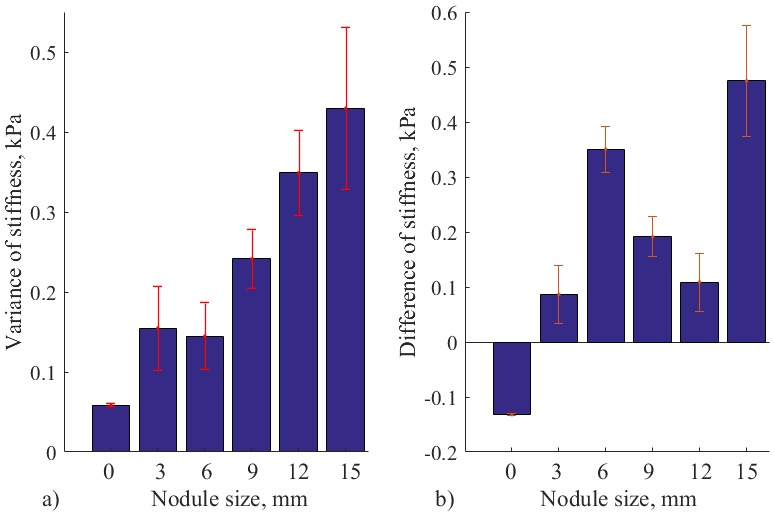
a) Variance of stiffness measurements for autonomous palpation, and b) Difference of stiffness for autonomous palpation and indentation based measurement. For silicone with no nodule, nodules of 3, 6, 9, 12 and 15 mm. Error bars show standard deviation for multiple trials.

The results of stiffness measurement for autonomous robotic palpation for different sizes of nodules are displayed on Fig 11. These results demonstrate how variable dynamics of the probe causes the modulation of stiffness. The recorded stiffness varies near the value of indentation based stiffness. Thus, the motion of the probe generates dynamic gain of stiffness that can be used to enhance the perception. To understand the nature of such response, variance and magnitude of the signal should be considered. Fig 12 visualizes the above parameters.

Mean variance of measured stiffness during autonomous palpation is displayed on Fig 12a). According to Wilcox rank test that was performed for pairwise comparison, there is a significant difference for silicone with no nodules and nodules of 12 and 15 mm (*Z* = −2.85, *p* < 0.05). Thus, temporal variability of the stiffness caused by the defined palpation behavior can enhance the perception of big-size nodules relatively to the probing instrument.

In addition to variance of the signal, it is important to see the difference between the real stiffness magnitude (indentation-based measurement) and the enhanced magnitude from autonomous palpation. The mean value of the magnitude from autonomous palpation is used. The values are represented on Fig 12b). It can be observed that the difference with is negative only for the case of soft silicone palpation with no nodule inside. Therefore, the stiffness calculated during autonomous exploration exceeds the stiffness threshold that was measured during simple force indentation test. It can be interpreted, that the static value of indentation-based stiffness is enhanced with some dynamic gain. Therefore, the presence of the nodule on a chosen location can be checked by using the obtained algorithm, and by comparing the measured stiffness with the indentation-based stiffness from the same location.

### Use of lateral and normal forces for the autonomous palpation

The motion of the probe or finger applies three-dimensional force to the phantom tissue. According to the correlation analysis in Section, lateral and normal forces are correlated. A final step of the the algorithm validation is the analysis of two components of the system—sinusoidal modulation of lateral force, and modulation of normal force caused by the autoregressive model. Here it is interesting to observe if it is really required to use both lateral and normal forces during robotic palpation. This is to apply both sinusoidal variation of the lateral force and the change of normal force according to AR model during one single palpation.

**Fig 13:**
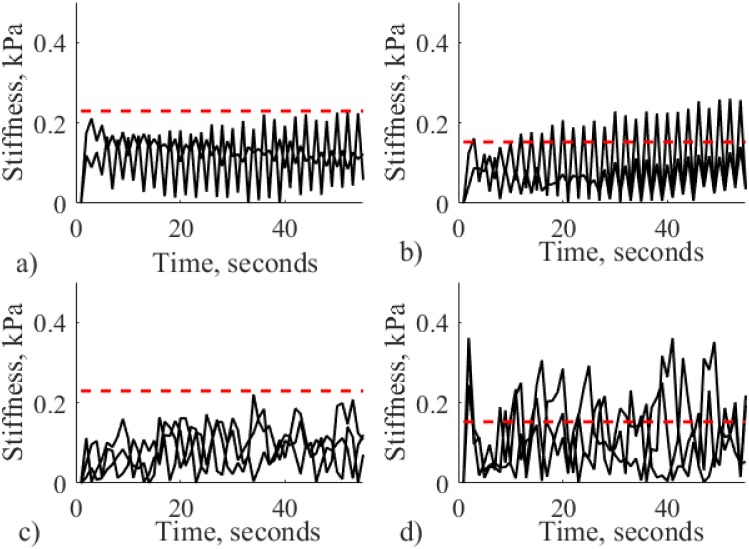
Stiffness obtained during validation experiments for nodules with 3 mm (a) and b)) and 9 mm diameter (c) and d)). Subfigures (a) and c)) show stiffness obtained using lateral force modulation of the robotic probe only. Subfigures (b) and d)) display stiffness for palpation using normal force generated by AR based palpation with no modulation of lateral force. Red dotted line shows stiffness measurements for indentation based palpation.

To validate this assumption, two behaviors are tested separately on the small nodules that might be difficult to detect—3 and 9 mm. In case the algorithm performs well for two separate behaviors, further tests are required to find the best approach. Alternatively, if the algorithm fails for two small nodules, then the approach outlined from human behavior (using both lateral and normal forces) outperforms the separate behavior, and further tests are not required. Lateral force modulation was performed with the fixed indentation of 5 mm, that corresponds to the depth of the embedded nodules. Similarly to the previous section, stiffness variability and difference from the indentation-based stiffness is evaluated. Fig 13 reflects the stiffness measurements for the two separate behaviors for each nodule.

**Fig 14:**
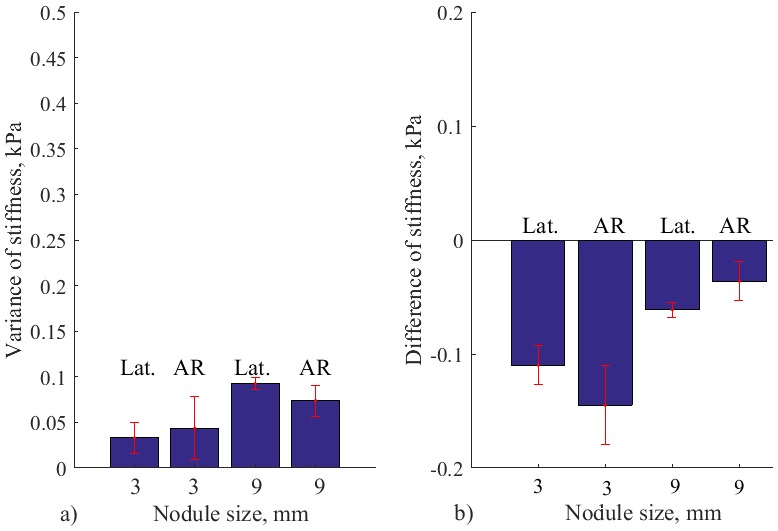
Results of validation studies. a) Variance of stiffness measurements and b) Difference of stiffness for autonomous palpation and indentation based measurement, for silicone with nodules of 3 mm and 9 mm. Results from validation studies for separate lateral force modulations (Lat.) and for AR based palpation with no lateral movement. Error bars show standard deviation for multiple trials.

The variation of stiffness and difference from the indentation-based palpation for the above experiment is reflected on Fig 14. It can be observed that separate lateral or normal force motions do not create modulations that exceed 0.09 kPa. While, the combined strategy produces the mean variance of 0.15 kPa and 0.24 kPa for diameters of 3 mm and 9 mm, respectively. In addition, it can be observed, that the magnitude of measured stiffness is not enhanced above the indentation-based palpation—the difference is negative. Therefore, it can be concluded that robotic palpation should be implemented using modulations of normal and lateral forces, according to the outlined human behavior.

### Nodule classification

**Fig 15:**
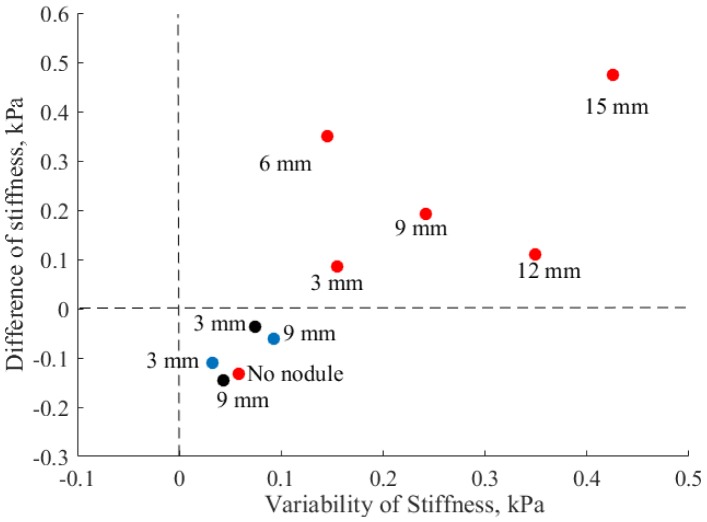
Variance and difference of stiffness of hard nodules and soft environment for robotic palpation, for three different strategies. Red points show the results of combined autonomous palpation, blue points correspond to validation of separate lateral motion, and black points to separate normal motion.

Fig 15 displays the variance and difference of stiffness during autonomous palpation in respect to the indentation-based for all robotic experiments to understand better the results obtained by the autonomous palpation, and to evaluate the separate components (lateral and normal modulations of forces). This graph can be used to evaluate the performance of robotic palpation based on the distance from the zero point. Zero denotes the stiffness that was measured during indentation based palpation. The closer the point is from zero, the less difference is between autonomous palpation and indentation based stiffness. In case the measurement is below the zero line threshold, then the performance is considered less efficient. Wilcox rank test shows a significant difference (*Z* = −3.74, *p* <0.05) between measurements displayed below the zero line and measurements above zero. The combination of high variability (from the mean stiffness) and high positive difference of stiffness (from the indentation-based palpation) demonstrates the effectiveness of autonomous robotic palpation.

It can be seen that autonomous palpation with both lateral and normal (AR model) motion enhances the stiffness of all nodules tested during first experiments (red points). Therefore, the dynamical interaction with the tissue containing harder areas increases the stiffness perception. Whereas, palpation of soft silicone without a nodule (red point “No nodule”) does not lead to stiffness enhancement. The silicone at the point of palpation is homogeneous, and the indentation-based stiffness already produces correct estimation of the measurement.

During the test of normal AR and lateral (blue dots) sinusoidal motion separately different results can be observed that supports the use of both lateral and normal AR motion simultaneously. Negative difference from indentation based palpation and low variability of stiffness does not indicate the presence of the nodules and can lead to misinterpretation by the user.

## Discussion

The focus of this work is on palpation of a defined local area to perform thorough tactile examination. However, the problem presented in this study should be considered together with the other challenges associated with robotic palpation. Mapping of the complex organ shape and control of the probe’s position in the viscoelastic environment is one of them. This problem can be solved in several different ways. One approach involves the use of tactile probes that measure both force and indentation [38]. Alternatively, it is possible to use virtual representation of the target organ [32]. Another area of studies that should be considered in conjunction with this work is the visualization of the obtained stiffness distribution from an organ. Array of tactile sensors can be considered as a good tool for visualization. However, this sensing solution does not always provide a good frequency response that makes it more suitable for static indentation methods. In the robotic implementation of force modulation strategy we demonstrate that the static indentation method can limit the possibility to dynamically enhance the tactile signal from the target area. Nevertheless, simple probing device with a spherical indenter, such as the one used in this studies also allows good visualization capabilities for dynamic data acquisition, as was demonstrated in [12]. In addition, such probe allows fast examination speed of the whole organ.

Local palpation techniques should be considered in conjunction with global examination of an organ that is used to detect areas of possible abnormalities. Due to complexity of the palpation environment and safety issues, tele-manipulated palpation is the most suitable approach for global examination. Therefore, it can be combined with autonomous palpation for selected areas to enhance the quality of the examination. To expand the validation of our approach, in future studies it is planned to consider robotic palpation of bigger nodules, as well as nodules of different shapes and orientation. Another important aspect is the real-time assessment of the system performance due to time constraints of the surgical operations. In our studies the identification of the nodule was observed within first thirty seconds of palpation. For further real-time analysis it is required to take into account global examination of an organ.

The findings presented in this work contribute to the general understanding of techniques and features of palpation for robotic applications. The summary of our findings, as well as other related works on human palpation behavior for robotic tactile examination, is shown in Table 2.

**Table 2:** Relevant work on human palpation studies for robotic applications.

Focus of study	Relevant findings	Reference
Palpation of hard nodules by humans	Force and velocity strategies are outlined for palpation of a continuous path	[39]
Global robotic palpation	Correctly chosen global, palpation pattern improves nodule identification	[40]
Evaluation of palpation skills of clinicians	Most efficient skills are: 1) Palpation of a coin with a paper cover; 2) Controlling, palpation pressure; 3) Discriminating changes in soft Tissues; 4) Integrating, skills with blindfold palpation.	[41]
Evaluation of learner’s, performance for breast palpation	Importance of global search palpation, and local palpation, pressure is highlighted	[42]
Software framework for robotic surgical tasks, segmentation, of hard inclusions	Results in the segmentation of hard inclusions helped to outline parameters such as sensitivity, specificity, duration, and safety.	[43, 44]
Vibration for robotic surgery	Dragging strategy of palpation is better combined with, vibro-tactile feedback, compared to pressure	[45]
Robotic palpation	Importance of interaction behaviour and internal stiffness control	[46]

In this study it was required to use a sensor with good technical parameters to perform validation and analysis of the proposed method. However, the advantage of force control strategies is that they can be used with simple probing devices, which are based on the indentation and force measurement principles. Therefore, there might be no need to use tactile devices that have complex mechanical design or sensing principle that is based on vibration or frequency. The work on the development of tactile probes for robotic palpation is still ongoing research. Nevertheless, various affordable solutions have been already developed to fulfill the requirements of the proposed system in terms of force range, sensitivity and response, such as [38, 47].

The work presented in this paper builds a base for the understanding local palpation behavioral strategies that can be used to improve the perception of non-homogeneous distribution in soft tissue. However, the studies can also be applied to the examination of viscoelastic environments for various other applications, such as the examination of the whole range of rubbers and silicones for industrial applications where a robot needs to perform sorting and manipulation of such materials, because we did not limit our participants to trained experts in any particular palpation technique.

## Conclusions

Three questions proposed in the beginning of this paper were investigated to study modulation of applied force during localized palpation. First, palpation behavior as demonstrated by humans is explored and formulated to understand the pattern of applied force. Based on the way humans modulate the applied force during tactile examination, an autoregressive model of finger movement pressure for local palpation was outlined. Humans perform sinusoidal modulation of lateral forces to enhance the perceived tactile information.

Second question was to investigate whether there exist a mathematical model that can represent the pattern of force modulations. The analysis of human behavior during palpation of hard nodules shows that the applied normal force can be modeled using the second order reactive model. This means that every next movement was planned according to the previously perceived information.

Then, to validate the abstracted palpation behavior and to answer the third question, autonomous robotic palpation is performed. Robotic behavior is created using recorded human demonstrations of tactile exploration. The robotic probe with a spherical indentation tip is used to autonomously palpate soft tissue silicon phantom. Thus, it was shown that the obtained model of modulation of the applied force enhances the perception of stiffness from a non-homogeneous environment and can be successfully used during robotic-based palpation. Also, we have demonstrated that it is necessary to use a combination of lateral and normal motion to achieve a more efficient exploration of the environment. The results have demonstrated that the applied behavior enhances the perceived stiffness of hard nodules with the help of an increased magnitude as well as variance of the perceived signal.
