# Correction: Palpation force modulation strategies to identify hard regions in soft tissue organs

**DOI:** 10.1371/journal.pone.0192259

**Published:** 2018-01-29

**Authors:** Jelizaveta Konstantinova, Giuseppe Cotugno, Prokar Dasgupta, Kaspar Althoefer, Thrishantha Nanayakkara

In the Funding section, the grant number from EPSRC MOTION is listed incorrectly. The correct grant number is: EP/N03211X/2.

There is an error in affiliation 4 for author Thrishantha Nanayakkara. Affiliation 4 should be: Dyson School of Design Engineering, Imperial College London, South Kensington Campus, London, United Kingdom.

## References

[1] Konstantinova J, Cotugno G, Dasgupta P, Althoefer K, Nanayakkara T (2017) Palpation force modulation strategies to identify hard regions in soft tissue organs. PLoS ONE 12(2): e0171706 https://doi.org/10.1371/journal.pone.0171706 28199349 10.1371/journal.pone.0171706 PMC5310901

